# Asymmetric supercapacitor of functionalised electrospun carbon fibers/poly(3,4-ethylenedioxythiophene)/manganese oxide//activated carbon with superior electrochemical performance

**DOI:** 10.1038/s41598-019-53421-w

**Published:** 2019-11-14

**Authors:** Muhammad Amirul Aizat Mohd Abdah, Nur Hawa Nabilah Azman, Shalini Kulandaivalu, Yusran Sulaiman

**Affiliations:** 1 Department of Chemistry, Faculty of Science, Universiti Putra Malaysia, 43400 UPM Serdang, Selangor Malaysia; 2 Functional Devices Laboratory, Institute of Advanced Technology, Universiti Putra Malaysia, 43400 UPM Serdang, Selangor Malaysia

**Keywords:** Supercapacitors, Energy

## Abstract

Asymmetric supercapacitors (ASC) have shown a great potential candidate for high-performance supercapacitor due to their wide operating potential which can remarkably enhance the capacitive behaviour. In present work, a novel positive electrode derived from functionalised carbon nanofibers/poly(3,4-ethylenedioxythiophene)/manganese oxide (*f*-CNFs/PEDOT/MnO_2_) was prepared using a multi-step route and activated carbon (AC) was fabricated as a negative electrode for ASC. A uniform distribution of PEDOT and MnO_2_ on *f*-CNFs as well as porous granular of AC are well-observed in FESEM. The assembled *f*-CNFs/PEDOT/MnO_2_//AC with an operating potential of 1.6 V can achieve a maximum specific capacitance of 537 F/g at a scan rate of 5 mV/s and good cycling stability (81.06% after cycling 8000 times). Furthermore, the as-prepared ASC exhibited reasonably high specific energy of 49.4 Wh/kg and low charge transfer resistance (*R*_ct_) of 2.27 Ω, thus, confirming *f*-CNFs/PEDOT/MnO_2_//AC as a promising electrode material for the future energy storage system.

## Introduction

The gradual depletion of fossil fuels and the demand of energy consumption have shown a tremendous effort in developing alternative energy storage devices such as fuel cells, lithium-ion batteries (LIBs), conventional capacitors and supercapacitors has been devoted to overcoming these energy crises. Among them, supercapacitors, also known as electrochemical capacitors or ultracapacitors have outperformed other energy storage devices by virtue of their superior electrochemical characteristics; high specific power, rapid charging/discharging rate and excellent long-term cycling stability^1^. Supercapacitors fill the gap between batteries and normal capacitors, which make them an important device for wide practical applications (hybrid vehicles, portable electronic devices and large-scale production)^2^.

Depending on the basis of the charge storage mechanism, supercapacitors are classified into two classes which are electrochemical double layer capacitors (EDLCs) and pseudocapacitors. In EDLCs, the electrical charges are stored via non-faradaic reaction at the electrolyte/electrode interface, while pseudocapacitors store charges by means of reversible redox reactions at the surface of the electrode. Carbon materials such as graphene^3^, graphene oxide (GO)^4^, carbon nanotubes (CNTs)^5^, carbon nanofibers (CNFs)^6^ and activated carbon (AC)^7^ are the common electrode materials for EDLC, and likewise, conducting polymers (CPs), transition metal oxides (TMOs), transition metal sulfides (TMSs), and transition metal nitrides (TMNs) are utilised as electrodes in pseudocapacitors. Among all carbon materials, CNFs have shown greater potential owing to their large specific surface area, excellent electrical conductivity, good mechanical stability, availability as well as environmental friendly^8^. The tunable diameter and pore size of CNFs can be achieved through a facile and practical combination of electrospinning and carbonisation^9^. However, the individual CNFs suffer from poor specific capacitance and specific energy. CPs including polyaniline (PANi)^10^, polypyrrole (PPy)^11–13^ and poly(3,4-ethylenedioxythiophene) (PEDOT)^14,15^ have drawn extensive interest in supercapacitors, but PEDOT has turned out to be the promising CP due to its excellent intrinsic conductivity and fast electrochemical kinetics^16^.

Manganese oxide (MnO_2_) is identified as an ideal pseudocapative-material in supercapacitors as it possesses theoretically high specific capacitance (*C*_sp_) (1370 F/g), wide operating potential, abundance in nature and non-toxicity characteristics^17^. Despite these advantages, challenges related to poor stability of PEDOT and low specific capacitance of MnO_2_ could hinder their practical supercapacitor application in the future. To overcome these problems, extensive efforts have been made in developing hybrid/asymmetric electrodes which combine the advantages of EDLCs and pseudocapacitors to further improve its capacitive performance. Moreover, the surface of CNFs can be altered by adding oxygen-containing functional groups (–COOH, –OH, and C=O), enhancing its surface wettability^18^ with better adhesion between active materials and CNFs^19^.

Recently, carbon-MnO_x_ composite fibers have been prepared by Pech and Maensiri^20^ using core-shell electrospinning and exhibited high *C*_sp_ of 213.7 F/g and good cycle durability (∼97%) over 1000 cycles. Śliwak and Gryglewicz^21^ reported a facile approach to synthesis MnO_2_/oxidised carbon nanofibers (MnO_2_/CNFox) and AC as positive and negative electrodes, respectively for asymmetric supercapacitor (ASC). The assembled ASC device with an extended potential window of 2.4 V possessed a remarkable specific energy of 24.8 Wh/kg (specific power at 100 W/kg) as well as excellent capacitance retention of 92.4% after 5000 cycles. Garcia-Torres and Crean^22^ reported the preparation of CB/CNT/MnO_2_/PEDOT:PSS composite fibers via wet spinning followed by chemical reduction using potassium permanganate (KMnO_4_) as reducing agent and displayed high *C*_sp_ of 351 F/g.

In this work, we reported the ASC design of functionalised-CNFs/PEDOT/MnO_2_ (*f*-CNFs/PEDOT/MnO_2_) composite and AC as positive and negative electrodes, respectively. The *f*-CNFs/PEDOT/MnO_2_ was fabricated through several steps; electrospinning, carbonisation, electrochemical functionalisation and electrodeposition of PEDOT and MnO_2_. The electrochemical performance was tested individually using three electrode configurations to determine the maximum operating potential achieved by each electrode. The asymmetric device was then assembled with a separator containing 1 M potassium chloride (KCl) electrolyte, and all electrochemical measurements were performed systematically. The synergistic effect contributed from both double layer capacitance and pseudocapacitance of *f*-CNFs/PEDOT/MnO_2_//AC has greatly improved its electrochemical performances including specific capacitance, specific energy and cycling stability.

## Results and Discussion

### Morphology characterisation

**Figure 1:**
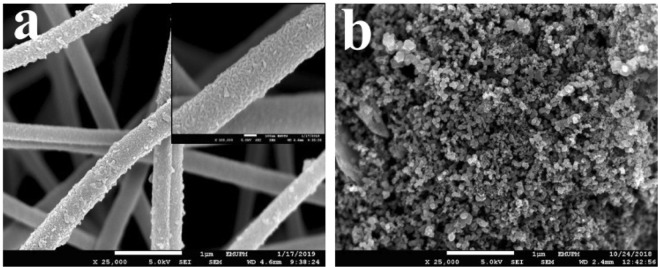
FESEM images of (**a**) *f*-CNFs/PEDOT/MnO_2_ (inset: the high-magnification of *f*-CNFs/PEDOT/MnO_2_) and (**b**) AC.

The surface morphology of the *f*-CNFs/PEDOT/MnO_2_ and AC electrodes were investigated through FESEM as shown in Fig. 1. In Fig. 1(a), the cross-linking structures of the as-prepared fibers are randomly oriented with smooth surface and beads-free. After the inclusion of PEDOT and MnO_2_ using the electrochemical approach, uniform growth of PEDOT and MnO_2_ nanoparticles on the *f*-CNFs surface can be observed without any aggregation. The presence of abundance oxygenated functional groups attached on CNFs can serve as nucleation sites for the growth of PEDOT and MnO_2_^23^ and providing better ions diffusion process from the electrolyte onto the electrode^11^. The *f*-CNFs/PEDOT/MnO_2_ has an average diameter of 390 ± 68 nm, which is slightly higher as compared with pure *f*-CNFs (354 ± 45 nm)^11^. The high-magnification FESEM image (inset of Fig. 1a) also proves that the coatings of PEDOT and MnO_2_ are relatively uniform on the fibrous networks. In Fig. 1(b), the AC electrode displays a homogenous and irregular carbon spheres morphology with an average diameter of 65 ± 12 nm. The void spaces between the AC particles provide more accessible surface sites that allow more contact surface between electrode and electrolyte ions^24^. Therefore, the unique morphology for both positive and negative electrodes can provide good charge propagation behaviour in ASC.

### Raman spectroscopy

**Figure 2:**
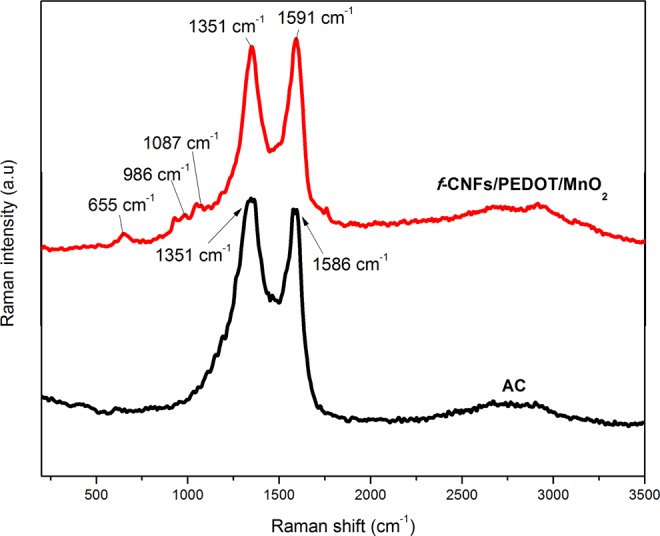
Raman spectra of as-prepared *f*-CNFs/PEDOT/MnO_2_ and AC.

Raman spectroscopy was used to study the functional groups that exist in both *f*-CNFs/PEDOT/MnO_2_ and AC electrodes as shown in Fig. 2. Two prominent peaks centred around 1351 and 1586 cm^−1^ in the AC spectrum, corresponding to the D and G bands, respectively. D band is related to the defect/disordered carbon structure (*sp*^3^) while G band originates from the crystalline graphitic layer (*sp*^2^). The D band (1351 cm^−1^) and G band (1591 cm^−1^) of the *f*-CNFs are also observed in the Raman spectrum of *f*-CNFs/PEDOT/MnO_2_. The presence of PEDOT in the spectrum is confirmed by three vibrational peaks; 986, 1087 and 1351 cm^−1^ which are associated with oxyethylene ring deformation, C–O–C deformation and C–C stretching vibration of PEDOT^25^, respectively. The intensity ratio (I_D_/I_G_) of *f*-CNFs/PEDOT/MnO_2_ is 0.85, which is slightly lower compared with AC (0.87), revealing a small number of defects in the sample^26^. In addition, a characteristic peak at 655 cm^−1^ is assigned to the stretching vibration of birnessite-type MnO_2_ ^27^.

### X-ray diffraction (XRD)

**Figure 3:**
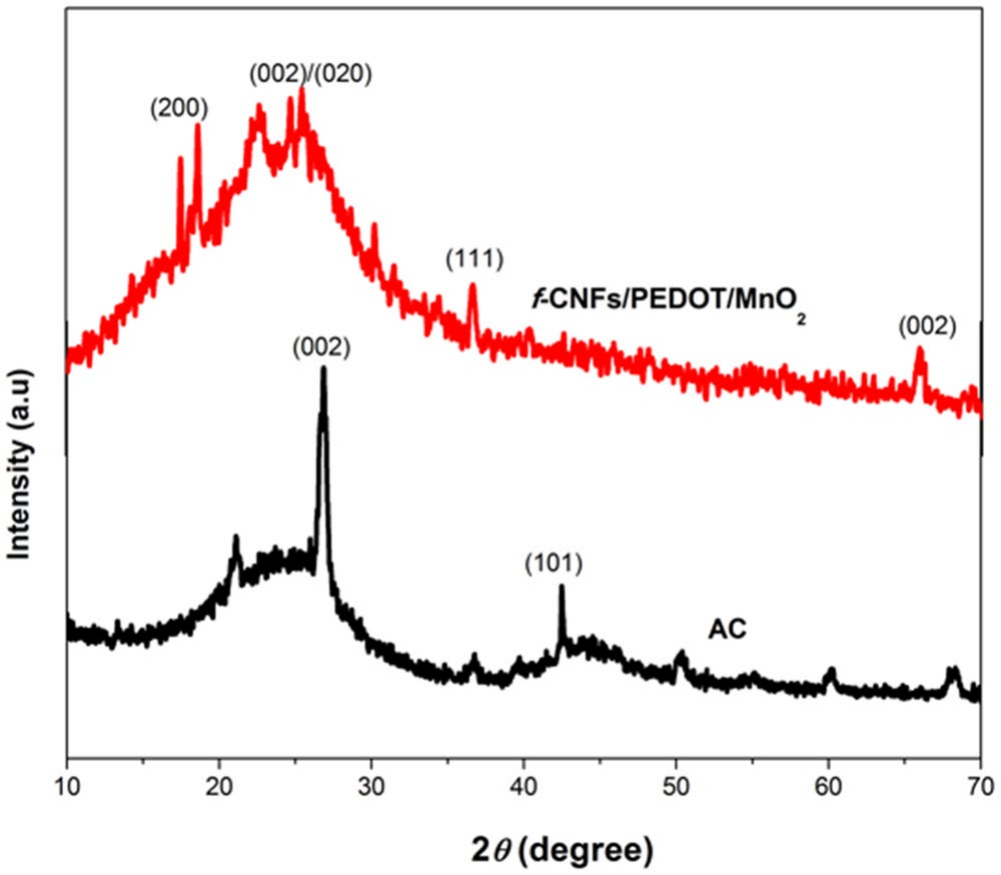
XRD diffractograms of *f*-CNFs/PEDOT/MnO_2_ and AC.

The crystallinity of *f*-CNFs/PEDOT/MnO_2_ and AC was investigated by XRD analysis, and the corresponding patterns are shown in Fig. 3. In the XRD pattern of *f*-CNFs/PEDOT/MnO_2_, a broad peak at 2*θ* = 25° can be indexed to the (002) diffraction plane of *f*-CNFs^28^ with an amorphous or low crystallinity carbon phase^29^. The presence of MnO_2_ nanoparticles was confirmed by two diffraction peaks positioned at 37° and 65° which can be identified as the (111) and (002) crystal planes of MnO_2_ (JCPDS Card. No. 24-0735)^30^. In addition, the sharp diffraction peaks at 2*θ* = 17° and 18° corresponded to the (200) plane of tetragonal α-MnO_2_ phase (JCPDS 072-1982). Furthermore, a low-intensity peak (2*θ* = 24°) which is overlapped with the broad peak of *f*-CNFs corresponding to the diffraction peak of PEDOT (020) with high-crystallinity feature^31^. As shown in Fig. 3, the typical characteristic of AC is visible at 2*θ* = 26° (002) and 43.5° (101)^32^ with a high degree of graphitic crystallinity. Notably, the additional peaks (2*θ* = 51° and 60°) appeared in the AC spectrum can be attributed to the bare ITO glass^33^.

### Electrochemical measurements

**Figure 4:**
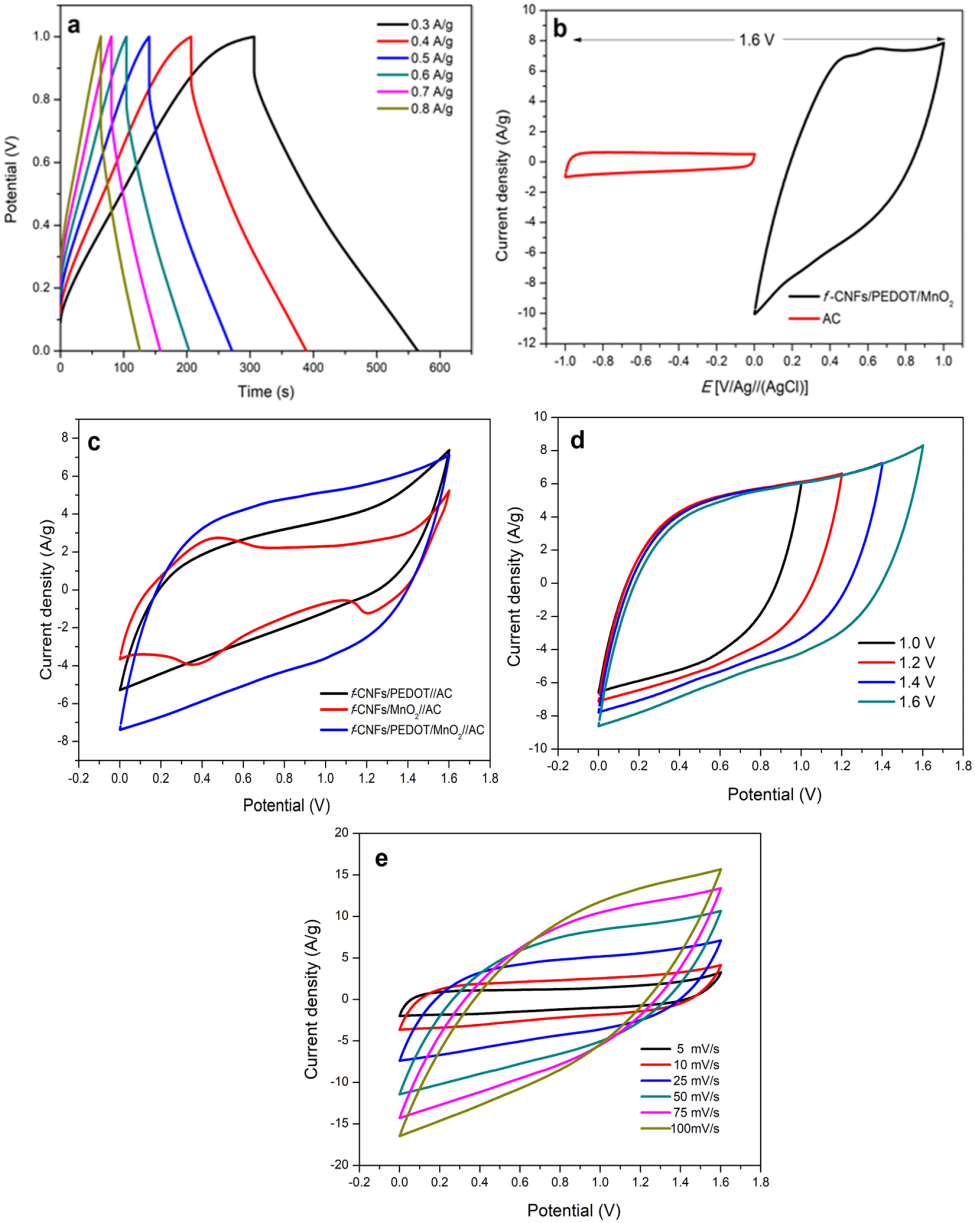
(**a**) GCD curves of *f*-CNFs/PEDOT/MnO_2_ at different current densities (0.3–0.8 A/g). (**b**) CV curves of *f*-CNFs/PEDOT/MnO_2_ and AC half cells at a scan rate of 25 mV/s. (**c**) CV curves of different asymmetric supercapacitors at a scan rate of 25 mV/s. (**d**) CV curves of asymmetric *f*-CNFs/PEDOT/MnO_2_//AC at different potential windows (1.0–1.6 V) using a scan rate of 25 mV/s. (**e**) CV curves of asymmetric *f*-CNFs/PEDOT/MnO_2_//AC at different scan rates from 5 to 100 mV/s in 1.0 M KCl electrolyte.

Figure 4(a) displays the charge-discharge behavior of *f*-CNFs/PEDOT/MnO_2_ at different current densities to understand its capacitive performance. The GCD curves exhibit nearly symmetrical triangular shape with a small deviation, which could be raised from the pseudo-faradaic reactions of PEDOT and MnO_2_ during the charging-discharging process. A noticeable *IR* drop at the initial portion of the discharge curves indicates the small internal resistance of the system at high charge-discharge current densities^34^. To evaluate the electrochemical performance of both *f*-CNFs/PEDOT/MnO_2_ and AC electrodes, CV measurements were carried out in a standard three-electrode setup using 1 M KCl as the electrolyte as shown in Fig. 4(b). The *f*-CNFs/PEDOT/MnO_2_ electrode was measured in the potential window of 0 to 1 V (vs. Ag/AgCl) and displays a quasi-rectangular shape, indicating the combination of both EDLC and pseudocapacitance behaviour^35^. However, a small hump observed at 0.56 V is attributed to the redox reaction of the oxygenated functional groups at the surface of the electrode^36^ and pseudocapacitance of PEDOT and MnO_2_. The *f*-CNFs/PEDOT/MnO_2_ electrode exhibits a larger enclosed CV area compared with AC, demonstrating higher charge capacity^37^ and higher specific capacitance^38^. For AC, the electrode contributes the potential window from −0.6 to 0 V (vs. Ag/AgCl) with symmetrical rectangular CV curve, indicating an ideal EDLC feature^39^ with good rate capability^21^. The *C*_sp_ for *f*-CNFs/PEDOT/MnO_2_ (442.50 F/g) and AC (58.89 F/g) are calculated based on Eq. (1):

1 $${C}_{sp}=\frac{1}{2m\times v\times \Delta V\,}\int I\,{\rm{dV}}$$

where *C*_sp_ (F/g) represents specific capacitance, *I* dV is the integrated area CV curve, *v* (Vs^−1^) is the potential scan rates, *m* (g) is the mass of sample, and dV (V) is the p, Δ*V* (V) is the potential window (CV) or potential drop during the discharging time (GCD) and *I* (A) is the applied current. The maximum potential window of the *f*-CNFs/PEDOT/MnO_2_//AC can be extended up to 1.6 V. Therefore, the charge of both electrodes need to be balanced in order to obtain a stable asymmetric supercapacitor via Eq. (2):

2 $$\frac{{m}_{+}}{{m}_{-}}=\frac{{C}_{-}\times \Delta {E}_{-}}{{C}_{+}\times \Delta {E}_{+}}$$

where *m* is the mass of electrode, *C* is *C*_sp_ of the respective electrode and ∆*E* is the operating voltage (0.6 V for negative and 1.0 V for positive electrode). The subscript of “+” and “−” relate to positive and negative electrodes. Figure 4(c) displays the CV curves of assembled *f*-CNFs/PEDOT//AC, *f*-CNFs/MnO_2_//AC and *f*-CNFs/PEDOT/MnO_2_//AC in the potential range of 0 to 1.6 V at a scan rate of 25 mV/s. It can be seen that *f*-CNFs/PEDOT/MnO_2_//AC shows a larger area of CV curve among other samples, suggesting high specific capacitance. Impressively, the assembled *f*-CNFs/PEDOT/MnO_2_//AC delivers the highest specific capacitance of 354 F/g compared with *f*-CNFs/PEDOT//AC (206 F/g) and *f*-CNFs /MnO_2_//AC (155 F/g). The CV curves of assembled *f*-CNFs/PEDOT/MnO_2_//AC asymmetric cell at different potential windows (1.0–1.6 V) are shown in Fig. 4(d). It can be seen that the ASC can work stably even at 1.6 V potential window and the quasi-rectangular of CV curves are retained, corresponding to good supercapacitive behaviour. Moreover, the specific capacitance increases from 287 F/g to 354 F/g over extended potential windows, which significantly improve the charge storage capacity of the composite^40^.

Figure 4(e) represents the CV of as-obtained ASC at different scan rates, ranging from 5 to 100 mV/s. At lower scan rates, the CV profiles exhibit rectangular-like shape and as the scan rate increases from 50 to 100 mV/s, the CV curves show some distortions from an ideal rectangular shape. This is due to the limitation of the electrolyte ions to diffuse into the active material and only capable to access at the outer surface of the material, leading to the decrease of specific capacitance^41^. The calculated specific capacitance of ASC can achieve 537, 459, 354, 269, 221 and 188 F/g at a scan rate of 5, 10, 25, 50, 75 and 100 mV/s, respectively. The prominent specific capacitance of ASC could be ascribed from the synergistic effect between *f*-CNFs/PEDOT/MnO_2_ and AC, where EDLC of *f*-CNFs and AC contribute to a larger ion-accessible surface area, while PEDOT and MnO_2_ possess excellent electrical conductivity and high specific capacitance, respectively.

**Figure 5:**
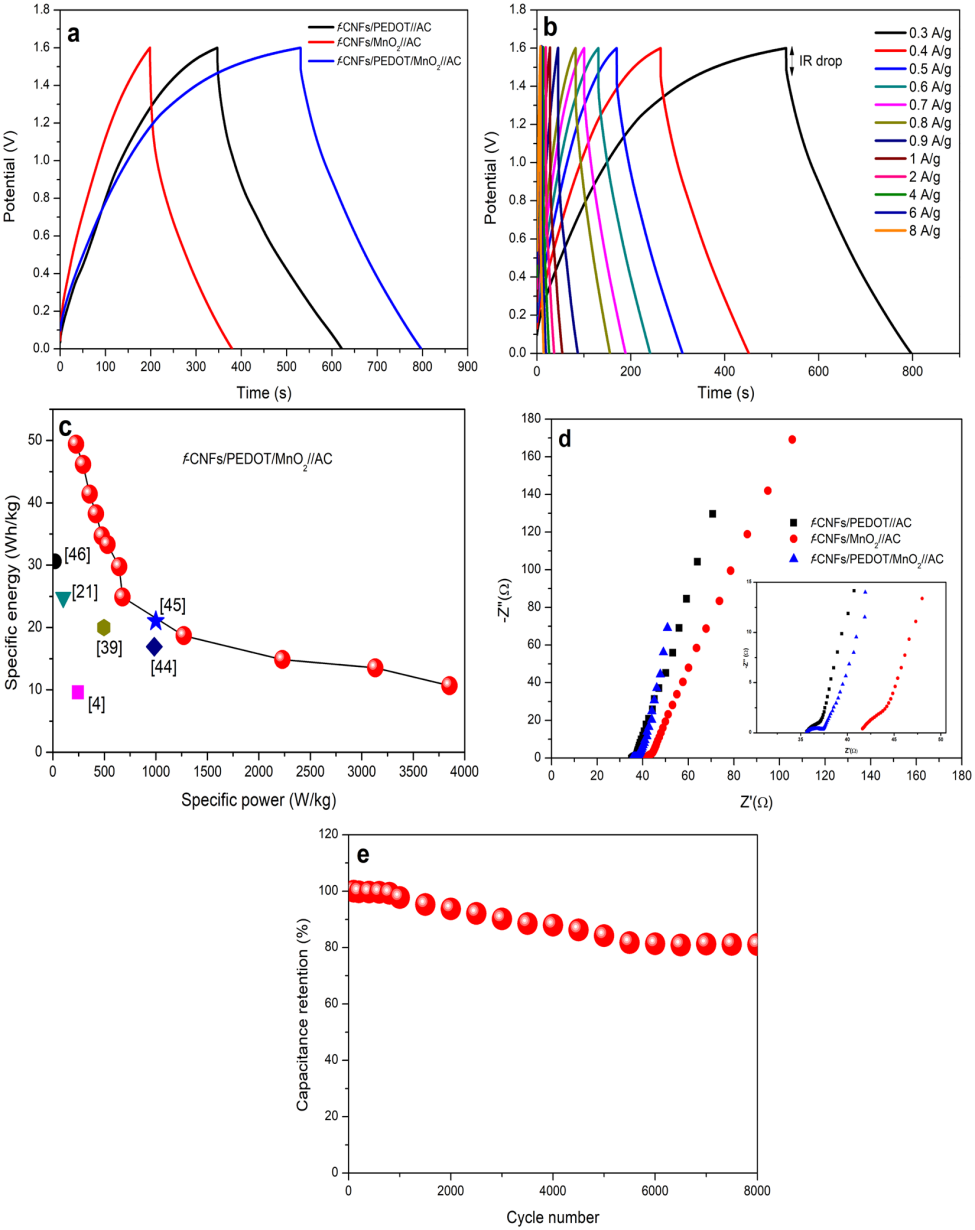
(**a**) GCD curves of different asymmetric supercapacitors at a current density of 0.5 A/g (**b**) GCD curves of asymmetric *f*-CNFs/PEDOT/MnO_2_//AC at various current densities (0.3–8 A/g). (**c**) Ragone plot of asymmetric *f*-CNFs/PEDOT/MnO_2_//AC. (**d**) Nyquist plot of different asymmetric supercapacitors with a frequency range of 0.01 Hz to 100 kHz. **(e**) The cyclic performance over 8000 cycles of asymmetric *f*-CNFs/PEDOT/MnO_2_ //AC electrode at a scan rate of 150 mV/s.

To further examine the electrochemical performance of ASCs, the GCD tests were carried out at a current density of 0.5 A/g as shown in Fig. 5(a). It can be clearly seen that *f*-CNFs/PEDOT/MnO_2_//AC electrode exhibits the largest charging-discharging time span, contributing to an excellent capacitive behaviour^42^. GCD measurement was also performed at various current densities; 0.3 to 8 A/g as presented in Fig. 5(b). Apparently, all GCD curves show a nearly triangular shape with a little deviation, corresponding to the signature of a redox-type storage mechanism^43^ with good electrochemical reversibility^24^. The specific capacitance of 148.07 F/g is obtained at a current density of 0.3 A/g. Figure 5(c) illustrates the Ragone plot (specific energy *vs* specific power) of *f*-CNFs/PEDOT/MnO_2_//AC asymmetric cell which exhibits maximum specific energy of 49.4 Wh/kg with a specific power of 224.02 W/kg at a current density of 0.3 A/g. Furthermore, the specific energy obtained is superior as compared to the reported values for PEDOT- and MnO_2_-based fibers for supercapacitor^4,21,39,44–46^.

The electrochemical properties of different asymmetric supercapacitors were further analysed using EIS measurements and their Nyquist plots are shown in Fig. 5(d). The plot consists of two regions: high and low frequency regions. At high frequency, the semicircle arc and the intercept of the real axis (*Z*’) indicate the charge transfer resistance (*R*_ct_) and equivalent series resistance (ESR), respectively. The straight line in the low-frequency region represents the Warburg impedance (*W*) for ion diffusion at the electrolyte/electrode interface. The *R*_ct_ of as-prepared *f*-CNFs/PEDOT/MnO_2_//AC, CNFs/PEDOT//AC and *f*-CNFs/MnO_2_//AC are 2.27, 1.2 and 2.74 Ω, respectively. A slightly higher *R*_ct_ of *f*-CNFs/PEDOT/MnO_2_//AC in comparison to *f*-CNFs/PEDOT//AC can be ascribed to the low conductivity of MnO_2_. Furthermore, the *f*-CNFs/PEDOT/MnO_2_//AC electrode shows the lowest ESR value of 35.58 Ω compared to *f*-CNFs/PEDOT//AC (35.63 Ω) and *f*-CNFs/MnO_2_//AC (41.28 Ω) as the ESR is attributed to the ionic resistance of the electrolyte, the resistance of the active material and contact resistance between the current collector and active material^47^. The nearly vertical line (close to 90°) at low frequency region of the *f*-CNFs/PEDOT/MnO_2_//AC indicates a characteristic of an ideal capacitive behaviour^48^. In addition, *f*-CNFs/PEDOT/MnO_2_//AC exhibits the shortest vertical line along the imaginary axis, implying rapid ion diffusion.

The cycling stability test of *f*-CNFs/PEDOT/MnO_2_//AC asymmetric cell was performed over 8000 CV cycles at a potential window of 1.6 V (Fig. 5(e)). Remarkably, the ASC device displays good cycling stability and retained 81.06% of its original capacitance after 8000 cycles. A good long-term cycling stability of ASC is mainly contributed from the superior mechanical strength of the *f*-CNFs and AC which effectively can enhance the cyclability during the charging/discharging process.

## Conclusions

In summary, a promising positive electrode derived from *f*-CNFs/PEDOT/MnO_2_ was successfully fabricated for asymmetric supercapacitor. By combining with AC as a negative electrode, the ASC device induces a strong synergistic effect which greatly enhanced its electrochemical performance. A well-adhered of PEDOT and MnO_2_ on the surface of the *f*-CNFs as well the highly porous morphology of AC was beneficial in providing ease ion-diffusion pathways at the electrolyte/electrode interface, thus can increase the specific capacitance. The assembled *f*-CNFs/PEDOT/MnO//AC could operate reversibly at a maximum voltage of 1.6 V, and displayed specific capacitance as high as 537 F/g, with good cycling stability (81.06%) after 8000 cycles. It also offers high specific energy of 49.4 Wh/kg at a specific power of 224.02 W/kg and low *R*_ct_, implying its good rate performance and enhanced conductivity. These outstanding results prove that *f*-CNFs/PEDOT/MnO//AC ASC can hold great potential for achieving high-performance supercapacitors.

## Experimental Section

### Materials

Indium tin oxide glasses (ITO, 7 Ω/sq) were obtained from Xin Yan Technology Limited. N, N-dimethylformamide (DMF) were supplied by Merck. AC, mesoporous carbon, polytetrafluoroethylene (PTFE), polyacrylonitrile (PAN, Mw = 150,000), silver wire (Ag, diam. 0.5 mm), 3, 4-ethylenedioxythiophene (EDOT, 97% purity), lithium perchlorate (LiClO_4_, ≥97% purity) and manganese (II) sulfate monohydrate (MnSO_4_•H_2_O) were purchased from Sigma-Aldrich. KCl and sulphuric acid (H_2_SO_4_) were acquired from Fisher Scientific. Acetone and ethanol were supplied from HmbG Chemicals and J. Kollin Chemicals, respectively. Deionised water (18.2 MΩ.cm) was used throughout the experiments. All chemicals were directly used as obtained without further purification.

### Preparation of *f*-CNFs/PEDOT/MnO_2_ (positive) and AC (negative) electrodes

The synthesis of *f*-CNFs/PEDOT/MnO_2_ composite was carried out using several steps: electrospinning, carbonisation, electrochemical functionalisation, electropolymerisation and electrodepositon sequentially. In a typical procedure, 10 wt% PAN solution in DMF under continuous stirring and a homogenous precursor solution was loaded into 5 mL syringe and ejected through a 15-gauge stainless steel needle by applying a voltage of 15 kV between needle and collector. The rotating collector covered by aluminium foil was placed 15 cm away from the tip of the needle with a flow rate of 1.0 mL/h and a rotating speed of 200 rpm. The electrospun PAN fibers were then oxidatively stabilised in a mild temperature of 280 °C under an air atmosphere at a heating rate of 1 °C/min. The stabilised fibers were then undergone carbonisation with a heating rate of 5 °C/min up to 800 °C for 2 h under nitrogen (N_2_) atmosphere. The attachment of oxygenated functional groups (–COOH, –OH, and C=O) on the CNFs surface was performed in 1.0 M H_2_SO_4_ via cyclic voltammetry for 2 h to obtain *f*-CNFs. The PEDOT film was electrochemically polymerised on *f*-CNFs at a constant potential of 1.1 V for 15 min, where Ag/Ag^+^ and Pt wire were served as a reference electrode and the auxiliary electrode, respectively. The electropolymerisation of EDOT was carried out in a non-aqueous solution consisting of 0.01 M EDOT and 0.1 M LiClO_4_ in acetonitrile. The MnO_2_ nanoparticles (0.05 M MnSO_4_•H_2_O solution) was then electrodeposited using cyclic voltammetry with a potential range between 0.1 and 1.0 V for 20 cycles, producing *f*-CNFs/PEDOT/MnO_2_ composite. Both electropolymerisation and electrodeposition were performed in a three-electrode configuration using a computer-controlled Autolab 101 potentiostat equipped with Nova 1.10 software. The AC electrode was prepared from an 80:10:10 (wt%) mixture of AC, mesoporous carbon and PTFE, where a few drops of ethanol were added into the mixture to form a slurry. The slurry was pasted onto ITO glass via and further dried in an oven at 50 °C. The mass loading of both *f*-CNFs/PEDOT/MnO_2_ and AC electrodes were about 0.5 mg/cm^2^ and 0.9 mg/cm^2^, respectively.

### Characterisations

The morphologies of *f*-CNFs/PEDOT/MnO_2_ and AC were examined using field emission scanning electron microscopy (FESEM, JEOL JSM-7600F). The structure and elemental composition of both positive and negative electrodes were identified using Raman spectroscopy (Alpha 300R, 532 nm Ar-ion laser) and X-ray diffraction (XRD, Shimadzu with Cu K∝ radiation (*λ* = 1.54 Å)

### Electrochemical test

Electrochemical measurements of the individual *f*-CNFs/PEDOT/MnO_2_ (positive) and AC (negative) were performed in a three-electrode configuration in 1.0 M KCl electrolyte. An Ag/AgCl and Pt wire were employed as a reference electrode and the auxiliary electrode, respectively. The CV of positive and negative electrodes was recorded at the voltage of 0–1.0 V and −0.6 to 0 V, respectively at a scan rate of 25 mV/s, respectively. The ASC two-electrode configuration was assembled by sandwiching both positive and negative electrodes together with a filter paper soaked in 1.0 M KCl as a separator. A series of electrochemical measurements, including CV, galvanostatic charge-discharge (GCD) and electrochemical impedance spectroscopy (EIS) was performed for ASCs. The CV analysis was performed from 0 to 1.6 V potential window at various scan rates (5–100 mV/s). GCD analysis was tested at a current density from 0.3–8 A/g. The *C*_sp_, specific energy (*E*) and specific power (*P*) of the ASC obtained from GCD curves were calculated according to Eqs (3–5):

3 $${C}_{{\rm{sp}}}=\frac{I\,\times \,\Delta t}{m\,\times \,\Delta V}$$

4 $$E=\frac{{C}_{sp}\Delta {V}^{2}}{2}$$

5 $$P=\frac{\Delta VI}{2m}$$

where *C*_sp_ (F/g) represents specific capacitance, Δt is discharge time (h), *m* (g) is the mass of active material (kg), Δ*V* (V) is the potential drop during the discharging time and *I* (A) is the applied current. EIS measurements were carried out in a frequency ranging from 0.01 Hz to 100 kHz at AC amplitude of 5 mV.
